# Characterization of a Bowman–Birk type trypsin inhibitor purified from seeds of *Solanum surattense*

**DOI:** 10.1038/s41598-021-87980-8

**Published:** 2021-04-21

**Authors:** Abhijeet P. Herwade, Sainath S. Kasar, Niraj R. Rane, Shadab Ahmed, Jaswinder Singh Maras, Pankaj K. Pawar

**Affiliations:** 1 Department of Biotechnology, Shivaji University, Kolhapur, MS 416004 India; 2 Department of Biochemistry, School of Life Sciences, Kavayitri Bahinabai Chaudhari North Maharashtra University, Jalgaon, MS 425001 India; 3 Biochemistry Division, Department of Chemistry, Savitribai Phule Pune University, Pune, MS 411007 India; 4 Institute of Bioinformatics and Biotechnology, Savitribai Phule Pune University, Pune, MS 411007 India; 5 Department of Molecular and Cellular Medicine, Institute of Liver and Biliary Science, New Delhi, 110070 India; 6 Department of Biochemistry, Shivaji University, Kolhapur, MS 416004 India

**Keywords:** Biochemistry, Biotechnology

## Abstract

A Bowman–Birk type trypsin inhibitor protein (SSTI) from seeds of the medicinal plant *Solanum surattense* was isolated, purified and characterized. SSTI showed a single band on SDS-PAGE corresponding to 11.4 kDa molecular weight. It is a glycoprotein (2.8% glycosylation) that differentially interacted with trypsin and chymotrypsin in a concentration-dependent manner. Its peptide sequence is similar to other Bowman–Birk type protease inhibitors found in *Glycine max* and *Phaseolus acutifolius*. The inhibitory activity was stable over a wide range of pH (1–10) and temperatures (10–100° C). Far-UV Circular Dichroism (CD) studies showed that SSTI contains β sheets (~ 23%) and α helix (~ 6%) and demonstrated structural stability at wide pH and high temperature. The kinetic analysis revealed a noncompetitive (mixed) type nature of SSTI and low inhibitor constant (Ki) values (16.6 × 10^−8^ M) suggested strong inhibitory activity. Isothermal titration calorimetric analysis revealed its high affinity towards trypsin with dissociation constant (K_d_) 2.28 µM.

## Introduction

Biotic stress induces the generation and accumulation of phenolic compounds and pathogenesis-related (PR) proteins which subsequently prevent an invasion of pests like insects and microbial pathogens^1^. Most of the plant PR proteins are acid-soluble, low molecular weight and protease enzyme inhibitors^2,3^. Protease inhibitors are mainly harbored by four plant families’ viz. Fabaceae, Gramineae, Leguminosae, and Solanaceae^4,5^. Plant protease inhibitors (PPIs) are categorized as serine, cysteine, threonine, aspartic and metallo-protease inhibitors based on their interaction with specific amino acid(s) present in the active site of protease^4^. On the basis of protein sequence and structural properties; PPIs are subdivided into 10 families’ as (1) Kunitz (serine) protease inhibitors; (2) Bowman–Birk (serine) protease inhibitors; (3) metallocarboxy protease inhibitors; (4) cysteine protease inhibitors; (5) cereal trypsin/*α*-amylase inhibitors; (6) potato type I; (7) potato type II protease inhibitors; (8) squash inhibitors; (9) serpins and (10) mustard trypsin inhibitors^6^. The molecular masses of Kunitz inhibitors are relatively high (~ 20,000 da) and possesses a single reactive site specific for trypsin. The Bowman–Birk inhibitors (BBIs) are a low molecular weight proteins (~ 9000 da), heat stable, double-headed and capable of inhibiting two serine proteases trypsin and chymotrypsin independently or simultaneously^7^.

PPIs are usually distributed in plant tissues; particularly; in the seeds and tubers along with leaves, flowers and fruits and protect the host from the attack of various insects^8,9^. Protease inhibitors (PIs) are produced in the plants’ endogenous defense system and expressed under various stress conditions such as attack by pathogens and insects, wounding and exposure to various environmental factors like salinity, heavy metals and temperature variations^5^. Most PIs interact with the specific active site of target proteases at the catalytic domain forming a stable protease inhibitor complex and there by inhibits the activity through competitive or non-competitive mode of action. The PIs are evolved as anti-metabolic proteins to control the growth of pathogens and insect pests as such proteins form requisite interaction with proteases of pathogens there by breaking down the supply of essential amino acids most needed by the pathogen for its survival^10^. The economically vital classes of pests like Lepidoptera, Diptera and Coleoptera use serine and cysteine type proteases in their digestive system. In the light of these facts, inhibitor(s) of serine proteases (trypsin/chymotrypsin) in insect diet is a wise approach to investigate its/their adverse effects on growth and development of economically important pests. The applications of inhibitors of proteases and α-amylases are well documented and they are considered to be one of the important tools in pest management strategies^11,12^. PPIs have various biological applications including apoptosis, hormone processing and blood clotting and PIs are also being studied on various parasitic or viral diseases including malaria, schistosomiasis, colds, flu', dengue etc^13^.

A vast number of plants belonging to family Solanaceae are reported for the presence of proteinaceous protease inhibitors. To quote a few such as *Ipomoea batatas* (sweet potato), *Solanum tuberosum* (potato), *Solanum palustre* and *Solanum nigrum* have been studied extensively^14–16^. As per our literature survey, there is no report on the presence of PI in *Solanum surattence* (Syn. *S. virginianum*)*,* a medicinally important member of family Solanaceae though this plant was previously reported for various biological applications^17–19^. Biochemical characterization and specificity of inhibition is an important step towards the discovery of an inhibitor and evaluate its/their potential. Present study reports isolation, purification and characterization of a Bowman–Birk type proteinaceous trypsin inhibitor from the seeds of *Solanum surattence.*

## Results and discussion

### Purified SSTI is a low molecular weight monomeric protein

Proteinaceous trypsin inhibitor (SSTI) was successfully isolated and purified from the seeds of *Solaum surattense*. About ninefold purification of SSTI was achieved through three steps protein purification method, (1) crude extract preparation; (2) partial purification of protein through ammonium sulphate precipitation followed by dialysis and (3) cation exchange column chromatography. The activity guided protein purification strategy was used for exact purification. The active concentrated fraction was utilized for further analysis. The drawn purification profile showed that about 0.56% yield of SSTI was obtained with 103.1 TIU/mg of trypsin inhibitory specific activity (Table 1). Previously, Ee et al.^20^ achieved a 0.54% yield of purified kunitz-type trypsin inhibitor from *Acacia victoriae* (Bentham) seeds. In couple of earlier studies on isolation of trypsin inhibitors from maize and sorghum achieved 4.96 and 5.78 fold purification respectively^21^. while about 12 fold purification of PI from the culture supernatant of S*treptomyces* spp. was obtained by Marathe et al.^22^ with a similar process as used for SSTI purification.

**Table 1:** Purification profile of SSTI.

Steps	Total protein (mg)	Total activity (TIU)	Specific activity (TIU/mg)	Yield %	Fold purification
Crude extract	860	9971	11.5	100	1
After dialysis	475.2	6078	13	55	1.2
Ion exchange chromatography	4.83	498	103.1	0.56	9

Total amount of protein extracted from 10 g of fine powder of dried seeds of *S. surattense.*

TIU (trypsin inhibitory activity unit) was defined as the decrease in 0.01 unit of absorbance at 410 nm.

**Figure 1:**
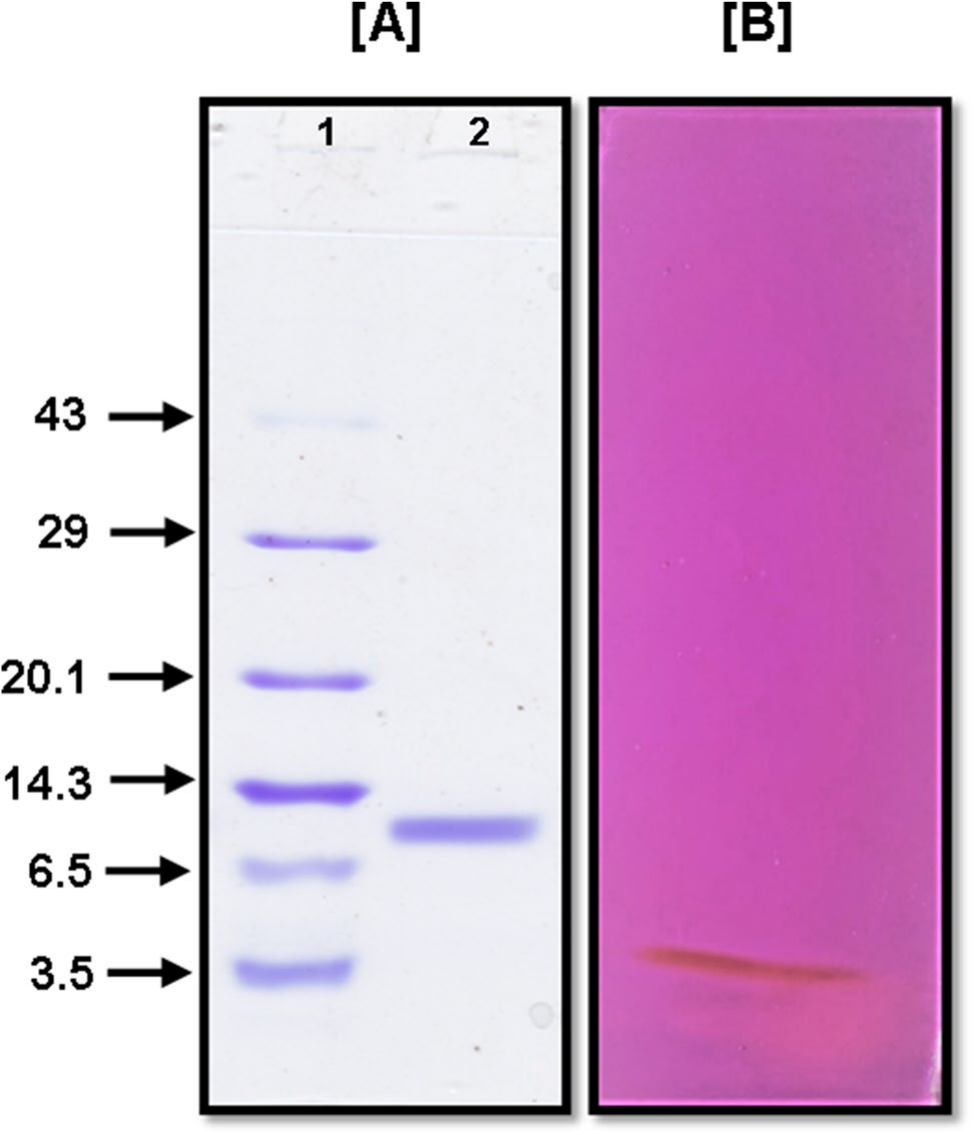
(**A**) SDS-PAGE profile (15%), analyses of purified SSTI from, *S. surattense* seeds, stained with Coomassie Brilliant Blue. (1) Standard protein markers, (2) purified fraction after cation exchange resin; (**B**) PAS staining of purified SSTI, indicating its glycoprotein nature (12% SDS-PAGE). Uncropped gel images are shown in Supplementary Figure S2A and B.

The homogeneity of isolated active protein fraction was analyzed by SDS–PAGE in presence of β-mercaptoethanol (reducing conditions) and a single protein band having a molecular mass of approximately 12 kDa was observed when compared with the standard molecular weight protein markers (Fig. 1A and Supplementary Figure S2A). This was further confirmed by Sephadex G-50 gel permeation chromatography where it was eluted as a single peak with 11.4 kDa molecular mass when log molecular masses of known proteins were plotted against the ratio of elution volume of protein peak and void volume (Figure S1). Single polypeptide chain and monomeric protein PIs of 18.9 kDa and 23.6 kDa molecular weight are earlier reported from the seeds of *Pithecellobium dumosum* and leaf extract of *Moringa oleifera* respectively^23,24^. Generally, the molecular mass range of PI is from less than 10–50 kDa and depends on the source^22^. Relatively low molecular weight of SSTI is in complete agreement with the hypothesis that the serine protease inhibitors from the plant are small proteinaceous entities with low molecular weight (3 to 25 kDa)^5^.

### SSTI is a glycoprotein

SSTI was proved to be a glycoprotein in nature as confirmed by reaction with Schiff’s reagent (PAS staining) on SDS-PAGE (Fig. 1B and Supplementary Figure S2B). The development of a magenta-colored single band was suggestive of the presence of glycan moiety in SSTI. Total carbohydrate content was quantitatively determined using phenol–sulfuric acid reagent and it was observed that SSTI contains 2.8% carbohydrate moieties in it. *Solanum tuberosum*, another member of family Solanaceae, is reported for glycosylated proteinaceous PI by Shah et al.^25^ while plants viz. *Swartzia pickellii*, *Peltophorum dubium*, *Echinodorus paniculatus* and *Acacia victoriae* from other families are also characterized by the presence of glycoprotein PI^26–29^.

### SSTI differentially interacted with different proteases

**Figure 2:**
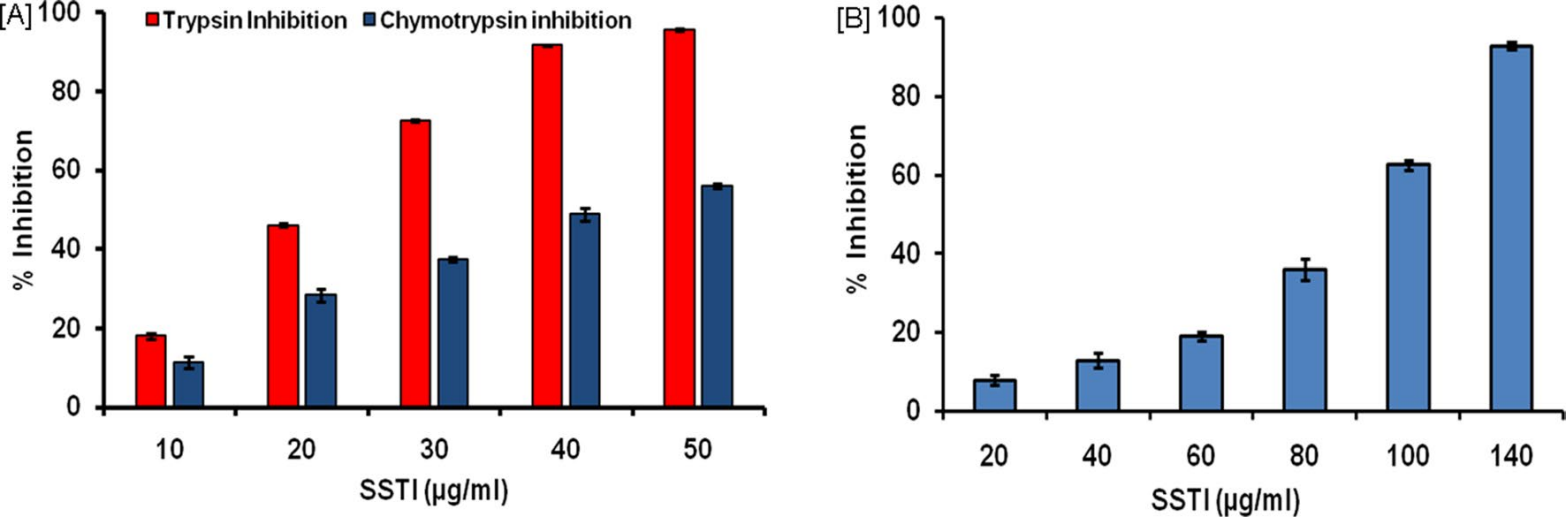
Concentration dependent inhibition of (**A**) Trypsin and Chymotrypsin (**B**) Gut proteases by Purified SSTI.

The concentration-dependent inhibition study involved the pre-incubation of various proteolytic enzymes (trypsin, chymotrypsin, proteinase k, and subtilisin) with increasing concentrations of SSTI (10–50 µg/ml). It was revealed that, SSTI could only inhibit trypsin and chymotrypsin among the four proteases. At 10 µg/ml concentration, SSTI inhibited the activity of trypsin and chymotrypsin by 18.14% (± 0.78) and 11.40% (± 1.54), respectively. The highest inhibition of trypsin and chymotrypsin activities i.e. 95.58% (± 0.4) and 55.91% (± 0.61) was observed at 50 µg/ml of SSTI (Fig. 2A). Purified SSTI was also assayed for its inhibitory activity against trypsin like protease enzyme isolated from gut of *Helicoverpa armigera*. Concentration dependent inhibition pattern was observed with *Helicoverpa armigera* Gut Protease (HGP). The concentration range of SSTI used for this study was 20–140 µg/ml, lowest inhibition (7.9% ± 1.29) and highest inhibition of HGP (92.9% ± 1.05) was recorded at 20 and 140 µg/ml of SSTI (Fig. 2B). However, SSTI did not have inhibitory potential with other proteases (proteinase k, subtilisin) even at the highest concentration used (50 µg/ml). A similar pattern of inhibition was reported for PI isolated from the seeds of *Pithecellobium dumosum*^23^. The PI from *Cassia leiandra* (ClTI) has been reported to have inhibitory potential against various enzymes like bovine trypsin, chymotrypsin, papain and porcine α-amylase^30^. Previously, the PIs are reported for inhibiting two or more proteases^31^.

### SSTI is a member of Bowman–Birk protease inhibitor family

Trypsin/chymotrypsin inhibitor from *S. surratence* was subjected to peptide mass fingerprinting. LC/MS/MS analysis of trypsin digested SSTI led to the identification of a Bowman–Birk type protease inhibitor. Four unique peptides matched with a Bowman–Birk domain-containing protein from *Glycine max* (Accession no. C6SVG7) with a significant coverage of 46.15%. Three other peptides matched with a Bowman–Birk type protease inhibitor from *Phaseolus acutifolius* (Accession no. P83311) with 41.25% coverage (Table 2).

**Table 2:** Mass spectroscopic profiles of SSTI protein from *Solanum surattence.*

Sample	Coverage %	Accession ID	Protein name	Observed peptide sequence	MW (da)
SSTI	46.15	C6SVG7	Bowman–Birk domain-containing protein OS = *Glycine max*		12.9
SSTI	41.25	P83311	Bowman–Birk type proteinase inhibitor OS = *Phaseolus acutifolius*		8.7

Peptides that matched with specific region of earlier kwon Bowman-Birk type protease inhibitors are highlighted in different colors and bold.

Bowman–Birk inhibitors from monocotyledons with molecular weight ~ 16 kDa have a double-headed structure of two separate functional inhibitory domains. The first reactive domain inhibits trypsin-like serine proteases mainly a positively charged residue (Arg or Lys) and the second domain with a large hydrophobic residue (Trp, Leu, Phe or Tyr) inhibits chymotrypsin-like serine proteases and a short hydrophobic residue (Val or Ile) inhibits elastase^32,33^. The peptide sequence of SSTI showed six amino acid residues (S-I-P-P-Q-C) which are observed to be conserved among many members of a Bowman–Birk type inhibitors^34^. This consensus sequence homology revealed that SSTI is a new member of the Bowman–Birk type protease inhibitor family with a unique cysteine framework.

### SSTI remains stable at a wide range of pH and temperatures

**Figure 3:**
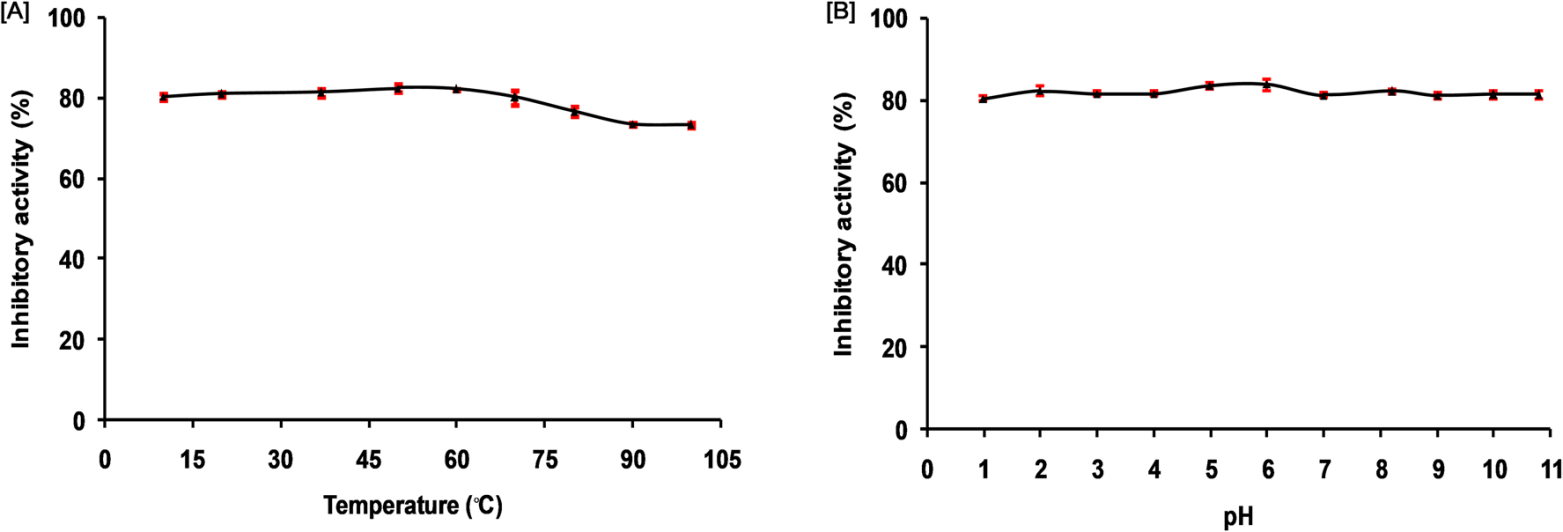
Stability of purified trypsin inhibitor (**A**) Temperature stability of purified SSTI incubation for 30 min at the indicated temperature, (**B**) pH stability of purified SSTI incubation at different pH for 30 min at 37 °C; The residual trypsin inhibitory activity was measured using BApNA as a substrate. In statistical analysis, one-way ANOVA was performed, followed by Tukey’s post hoc test. Statistical data are significant at *P* < 0.001.

The thermal stability study showed that SSTI remained stable between 10 and 70 °C and experienced moderate loss in trypsin-inhibition activity at higher temperatures, at 100 °C SSTI showed nearly 11% loss in trypsin inhibition capability (Fig. 3A). SSTI maintained substantial inhibitory potential against target trypsin enzyme up to 100 °C. It is observed by earlier researchers that protease inhibitors isolated from seeds are quite thermostable up to 100 °C for example; inhibitors isolated from seeds of *Phaseolus aureus* Roxb. (Mung bean), *Pithecellobium dumosum* and *Rhynchosia sublobata* (Schumach.)^23,35,36^. In pH stability study, we observed that SSTI has no remarkable loss in trypsin inhibition between the pH range of 1.0–10.8 and it retained its functional strength at extreme pH conditions (acidic as well as alkaline conditions) (Fig. 3B). This type of broad pH stability is reported in Bowman–Birk family trypsin inhibitors isolated from seeds of *Canavalia lineate* seeds, *Glycine soja* (wild Soja) and *Phaseolus aureus* Roxb. (Mung bean)^36–38^ and kunitz-type inhibitors from *Peltophorum dubium* seeds^27^. Generally, the stability of a BBI type trypsin inhibitors towards physical (temperature, pH) and chemical (reducing agents) denaturants is associated with the presence of several disulfide bonds^33,39^.

### CD spectroscopy

**Figure 4:**
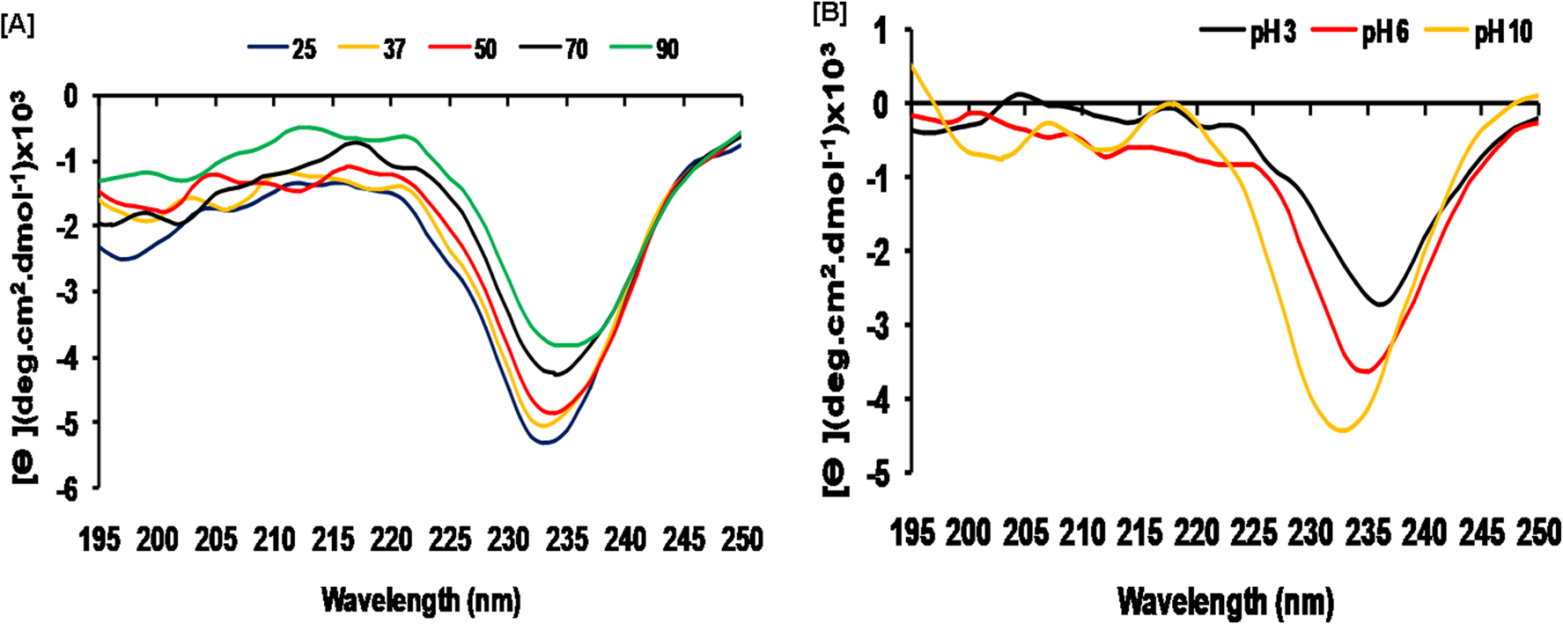
CD studies of SSTI (**A**) Far UV CD spectra (195–250 nm) as function of different temperature incubate for 30 min and (**B**) Different pH incubate for 30 min.

CD spectra have been used to analyze the secondary structure of SSTI at different pH and temperatures. Far-UV CD spectra of SSTI are presented in Fig. 4; it exhibited an intense negative peak at 225–240 nm and no positive ellipticity. According to this study, SSTI is composed of 23% β-sheets and 6.03% α helix. The conformational stability studied by CD spectra analysis demonstrated the thermal and pH stability of SSTI structure. Increasing temperature and extreme pH resulted no progressive loss of the conformational pattern of SSTI and retain the structural stability (Fig. 4A,B). These observations are in supportive to with our observations about SSTIs stability at higher temperatures and wide range of pH. Previously, significant structural stability in broad pH and high temperatures is reported for other Bowman–Birk protease inhibitor^34,39^. Circular dichromism study confirmed that the protein contained high β-sheet structures; a similar pattern of secondary structural elements was reported for PI isolated from *Rhynchosia sublobata* (Schumach.) Meikle seeds^35,39^.

### SSTI shows noncompetitive (mixed) type of trypsin inhibition

**Figure 5:**
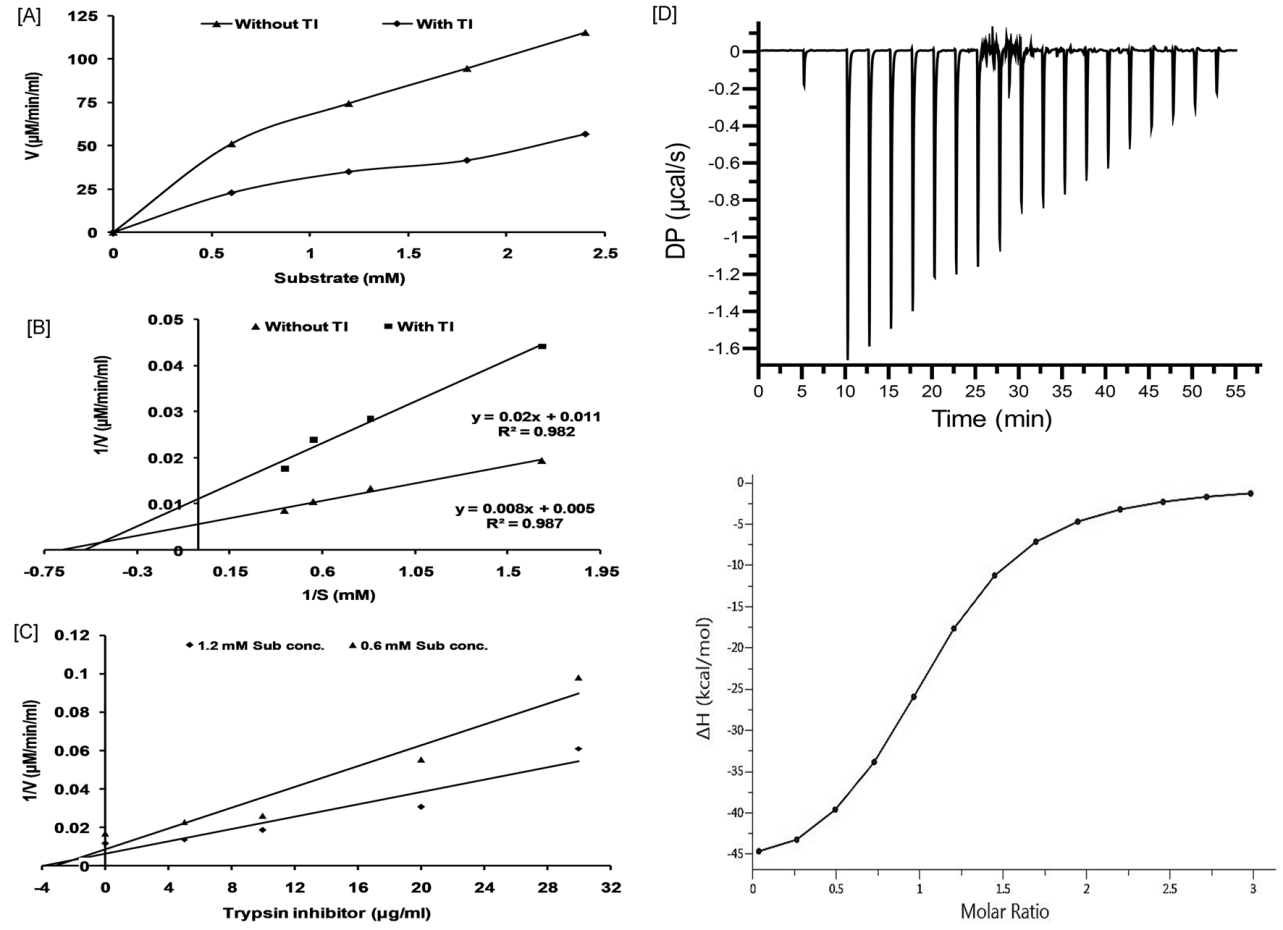
Kinetic analysis of purified SSTI. Noncompetitive (mixed) type of inhibition of bovine trypsin (**A**) Michaelis–Menten kinetics of bovine trypsin in presence and in absence of SSTI. (**B**) Lineweaver–Burk plot of 1/[V] versus 1/[S] of bovine trypsin in presence and in absence of SSTI. (C) Dixon’s plot of 1/[V] versus [I] of bovine trypsin in presence of different concentrations of SSTI at two different concentrations of BApNA. The Ki value for SSTI was 16.6 × 10^−8^ M. (D) Interaction by Isothermal titration of purified SSTI with bovine trypsin.

To understand the inhibition mechanism of SSTI, data generated by the kinetic study was analyzed through Michaelis–Menten kinetics, Lineweavere Burk plot and Dixon plots by following a single substrate-single inhibitor approach. According to Michaelis–Menten plot, the inclusion of SSTI affected the velocity of trypsin catalyzed proteolysis (Fig. 5A). We have observed that Km and Vmax in absence of inhibitor were found to be 1.6 mM and 200 U (μM/min/ml) respectively. However, in presence of SSTI, the Km and Vmax were observed to be 1.81 mM and 90.9 U respectively (Fig. 5B). These findings are suggestive of noncompetitive (mixed) type inhibition by SSTI. The inhibition constant (Ki) for purified SSTI obtained from the Dixon plot, was 2 μg/ml equivalent to 16.6 × 10^−8^ M (calculated based on the molecular weight of PI) (Fig. 5C). A similar type of inhibition was reported earlier with PI obtained from seeds of *Tamarindus indica* and *Entada scandens* with Ki values of 1.7 × 10^−9^ M and 4.9 × 10^−9^ M, respectively^40,41^.

### Interactions of SSTI with trypsin as evaluated by isothermal titration calorimetry

Isothermal titration calorimetry is a powerful and very sensitive technique based on the simple measurement of heat changes (q) as a consequence of the biomolecular interaction. When a molecule goes from free-state to bound state it is invariably associated with either redistribution or formation of non-covalent bonds which results in heat changes and ITC makes note of these changes in terms of differential power needed to maintain the zero temperature difference between the sample and reference cell. ITC data analysis revealed that the isolated inhibitor is tightly bound to trypsin with K_d_ value of 2.28 µM (Fig. 5D). Binding actions are characterized by a largely favorable enthalpy with ΔH value of − 80 kcal/mol and entropy change (− TΔS) value of 72 kcal/mol. Using this technique, we have plotted a signature plot for the same which revealed the interactions having significant hydrogen bonding and van der Waals interactions.

## Methods

The study on plant material including the collection of seeds had complied with relevant institutional, national and international guidelines and legislation.

### Plant Materials

The plant materials for the present study were collected from Kagal MIDC, Kolhapur, Maharashtra, India (16° 37′ 08.2" N, 74° 21′ 12.4" E). The specimens were then identified by Dr. Manoj M. Lekhak, Assistant Professor, Department of Botany, Shivaji University, Kolhapur, Maharashtra, India and the voucher specimens (001) deposited in the Herbarium of Department of Botany, Shivaji University, Kolhapur, Maharashtra, India. The plant materials were collected with the permission and guidance by the local authority at Kagal MIDC, Kolhapur, Maharashtra, India. An official letter of permission was submitted and approved by the authorities prior to sample collection. The seeds were used for experimentation after washing and cleaning and kept in clean, dried environment at room temperature.

### Isolation and purification of trypsin inhibitor (SSTI) protein from the seeds of *S. surattense*

Ten gram fine powder of dried seeds of *S. surattense* (Syn. *S. virginianum*) was taken to prepare a crude extract of trypsin inhibitor. The extraction of proteins was performed as per the method described by Kasar et al*.*^42^ with slight modifications. The crude extract was centrifuged at 4712×*g* for 20 min at 4 °C and the resultant supernatant was used for ammonium sulphate precipitation (80%). The precipitated pellets thus obtained were used for dialysis followed by lyophilization. Further, concentrated dialyzed proteins were subjected to CM-Cellulose pre-packed column (20 cm × 3 cm) pre-equilibrated with citrate buffer (pH 5.3, 0.02 M) for ion exchange chromatography. The fractions showing the highest trypsin inhibition were pooled and concentrated using Amicon Ultra-15 centrifugal filter units (Sigma Aldrich, St. Louis, MO, USA). Total protein content at each step of purification was determined as per the method described by Lowry et al.^43^.

### Studies on protease inhibitory activity of SSTI

Protease inhibitory potential of SSTI was evaluated on different proteases like Bovine pancreatic trypsin, Chymotrypsin, Proteinase K, Subtilisin and *H. armigera* gut protease (gut homogenate was used as protease source).

Trypsin inhibitory activity assay was performed using bovine pancreatic trypsin and its synthetic substrate Benzoyl-Arginyl-*p*-Nitro-Anilide (BApNA) by a following method described by Erlanger et al.^44^ In this, different concentrations (5 to 50 μg/ml) of purified *S. Surattense* trypsin inhibitor (SSTI) were added to 30 µl of trypsin (300 μg/ml) followed by addition of 0.1 M Tris–HCl buffer (pH 8.0 containing 20 mM CaCl_2_) to achieve the volume of enzyme and its inhibitor mixture to 300 μl and incubated at 37 °C for 15 min. Then, 500 μl BApNA (1.25 mM) was added in pre-warmed mixture and incubated at 37 °C for 15 min. After incubation, reaction was stopped by adding 200 μl of 30% (v/v) glacial acetic acid and liberated p-nitroaniline was monitored at 410 nm. One Trypsin inhibitor unit (TIU) was defined as amount of inhibitor needed to decrease the absorbance by 0.01 units at 410 nm under the trypsin inhibition assay^45^.

Besides trypsin inhibition, Chymotrypsin, Proteinase K, Subtilisin inhibitory activity of SSTI was also assayed using Caseinolytic approaches as reported by Marathe et al.^22^ with slight modifications. Protease inhibitor 50 μg/ml was preincubated with different proteases at 37 °C for 15 min. After preincubation, 500 μl of 0.1% casein was added and incubated at 37 °C for 15 min. Then, the reaction was terminated by adding 300 μl of 10% trichloroacetic acid (TCA) solution. The samples were centrifuged and the supernatant was measured at 280 nm using a UV–Visible spectrophotometer (Shimadzu, Japan).

For extraction of crude protease, the mid gut tissues were dissected carefully from the fourth or fifth instar larvae of *H. armigera* as described by Giri et al.^46^ The excised midgut tissue samples were collected and homogenized in chilled 0.1 M Tris–HCl buffer (pH 8.8). The suspension was centrifuged at 4 °C for 20 min at 4712×*g*. The supernatant was collected and assayed for protease enzymic activity as described earlier^45^.

### Determination of molecular weight of SSTI using SDS-PAGE and gel permeation chromatography

The molecular mass of isolated SSTI was determined on polyacrylamide gel (15%) under reducing conditions as described by Laemmli^47^. Trypsin inhibitory active fractions were separated by using SDS-PAGE and visualized by staining with Coomassie Brilliant Blue (CBB-R-250) for 60 min followed by distaining with methanol:acetic acid:water (30:20:50). The molecular weight of purified SSTI protein was calculated by comparing its relative position with molecular weight markers. Further, a molecular weight of purified SSTI was also confirmed by using size exclusion chromatography on a pre-equilibrated Sephadex G-50, Tris–HCl (20 mM, pH 7.5) column (105 cm × 1.8 cm).

### Determination of carbohydrate content and glycoprotein staining analysis

Total carbohydrate content present with purified SSTI was estimated by phenol sulphuric acid method by using glucose as a standard^48^. Phenol–sulphuric-acid-positive carbohydrates were expressed as a percentage of glucose equivalent as determined from a standard calibration curve using 0.1 mg/ml stock solution of glucose. Glycoprotein nature of purified SSTI was determined by Periodic Acid Schiff (PAS) staining as described by Kasar et al.^43^.

### Mass spectrometry analysis

100 µg equivalent protein was subjected to reduction, alkylation followed by digestion for 16–20 h at 37 °C using sequencing-grade modified trypsin: proteins (1 µg:20ug w/w). The samples were desalted and subjected to LC–MS/MS analysis. The peptides were eluted by a 3–95% gradient of buffer B (aqueous 80% acetonitrile in 0.1% formic acid) at a flow rate of 300 nl/min for about 60 min on a 25-cm analytical C18 column (C18, 3 μm, 100 Å). The peptides were ionized by nano-electrospray and subsequent tandem mass spectrometry (MS/MS) on a Q-Exactive Plus was performed and analyzed using a mass spectrometer with the collision-induced dissociation mode with the electrospray voltage was 2.3 kV. Analysis on the orbitrap was performed with full scan MS spectra with a resolution of 70,000 from m/z 350 to 1800. The MS/MS data were analyzed by Proteome Discoverer (version 2.3, Thermo Fisher Scientific, Waltham, MA, United States) using the Self curated data base of the protease inhibitors (The Uniprot data base). Proteins with FDR > 0.01 and *p* value > 0.05 were only used for analysis^49^.

### Thermo-stability and pH tolerance

The impact of pH and temperature on the inhibitory activity of SSTI was evaluated according to Dias et al*.*^30^ To study the thermal stability of SSTI, it was incubated at varying temperatures ranging from 10 to 100 °C (± 1 °C) for 30 min. After incubation at various temperatures aliquots were cooled and activity was measured against the bovine trypsin. Similarly, to check pH stability of SSTI, aliquots of SSTI were incubated with different pH buffers (0.05 M), namely KCl–HCl buffer (pH 1–3), citrate buffer (pH 4–6), phosphate buffer (pH 7), Tris-Cl buffer (pH 8–9) and Glycine–NaOH buffer (pH10-11) for 30 min at 37 °C and activity was measured against the bovine trypsin.

### Circular dichroism (CD) spectroscopy

All far-UV CD spectra (195–250) were obtained under nitrogen atmosphere at 25 °C and recorded on a JASCO J-715 (Jasco Inc., Easton, MD, USA) spectropolarimeter using solutions of a protein concentration 1 mg/ml in the quartz cuvette with a 1-mm optical path. All spectra were generated by taking an average of three scans and were corrected for the respective blanks. Results are expressed as molar ellipticity, [Θ] (deg cm^2^/dmol), based on a mean amino acid residue weight (MRW)^50^.

### Analysis of kinetic behavior of SSTI with trypsin

The kinetic behavior of SSTI during its interaction with trypsin was studied with different concentrations of the inhibitor [I] and substrate [S]. A Lineweaver–Burk graph was plotted and analyzed to determine the nature of inhibition. For this, 30 µl of trypsin (0.3 mg/ml) was incubated with different concentrations of substrate ranging from 0.6 to 2.4 mM BApNA (at an interval of 0.6 mM). The reaction was stopped by the addition of 200 µl of 30% (v/v) acetic acid. The liberated compound p-nitroaniline was measured at 410 nm. The Km and Vmax values of trypsin were determined by a double reciprocal plot of substrate concentration v/s velocity in the presence and absence of SSTI. The Ki value was determined through Dixon’s plot by assaying trypsin with various concentrations of SSTI at two substrate concentrations (0.6 and 1.25 mM) under standard assay conditions^51^.

### Isothermal titration calorimetry study of SSTI

A MicroCal PEAQ-ITC instrument (Malvern Panalytical Ltd., U.K.) was used to determine the thermodynamic properties of a chemical or physical equilibria resulting from the titration of 0.1 mM SSTI with 1 mM bovine trypsin in titration buffer (10 mM Tris at pH 7.8). Nineteen injections of 2 μl SSTI injected sequentially into a titration cell at 37 °C containing 300 μl trypsin. Each injection was delivered after every 200 s. The MicroCal PEAQ-ITC Analysis Software was used to extract the data file using the “One Binding Site” fitting model. Further analysis of kcal mol^−1^ of injection of ligand vs molar ratio graph enabled calculation of affinity constants (K_d_), binding enthalpy changes (ΔH) and binding stoichiometry (n) by fitting the integrated titration peaks.

### Data analysis

Data from the protease inhibition assay, SSTI stability study were subjected to statistical analysis, one-way ANOVA followed by Tukey’s post *hoc test*. The *p* value was considered to be greater than the α-value (level of significance 0.05, i.e. *p* < 0.05).

## Conclusion

In present investigation a functional glycoprotein SSTI was isolated and purified from the seeds of *S. surattense,* a medicinally important member of the family Solanaceae. SSTI is identified as a Bowman-Birk type protease inhibitor family with a unique consensus sequence. It was temperature-pH tolerant protein and has inhibitory efficacy with trypsin and chymotrypsin. Circular dichroism confirmed that the protein contained high β-sheet structures. The monomeric low molecular weight SSTI was revealed to be a noncompetitive (mixed) type inhibitor of trypsin. In future, a detailed molecular interaction, protein engineering and structural analysis study will be helpful to throw a light on its actual mechanism of inhibition.

## Supplementary Information

Supplementary Information
