# Supplementary material for: Characterization of a Bowman–Birk type trypsin inhibitor purified from seeds of Solanum surattense
Source: Sci Rep. 2021 Apr 21;11:8648. doi: 10.1038/s41598-021-87980-8 (PMC8060351; doi:10.1038/s41598-021-87980-8)
Supplement: Supplementary file 1 — Supplementary Information [file 41598_2021_87980_MOESM1_ESM.docx]

**Characterization of a Bowman-Birk type trypsin inhibitor purified from seeds of *Solanum surattense***

Abhijeet P. Herwade^1^, Sainath S. Kasar^1,2^, Niraj R. Rane^3^, Shadab Ahmed^4^, Jaswinder Singh Maras^5^  and Pankaj K. Pawar^6*^

^1^ Department of Biotechnology, Shivaji University, Kolhapur, (MS), India-416004

^2^Department of Biochemistry, School of Life Sciences, Kavayitri Bahinabai Chaudhari North Maharashtra University, Jalgaon, (MS), India-425001

^3^Biochemistry Division, Department of Chemistry, Savitribai Phule Pune University,

Pune, (MS), India-411007

^4^Institute of Bioinformatics and Biotechnology, Savitribai Phule Pune University,

Pune,(MS), India-411007

^5^Department of Molecular and Cellular Medicine, Institute of Liver and Biliary Science, New Delhi, India-110070

^6^Department of Biochemistry, Shivaji University, Kolhapur, (MS), India-416004

***Corresponding author**

Dr. Pankaj K. Pawar,

Associate Professor,

Department of Biochemistry,

Shivaji University, Kolhapur, (MS), India-416004

Email: [pkp.biochem@unishivaji.ac.in](mailto:pkp.biochem@unishivaji.ac.in)

**
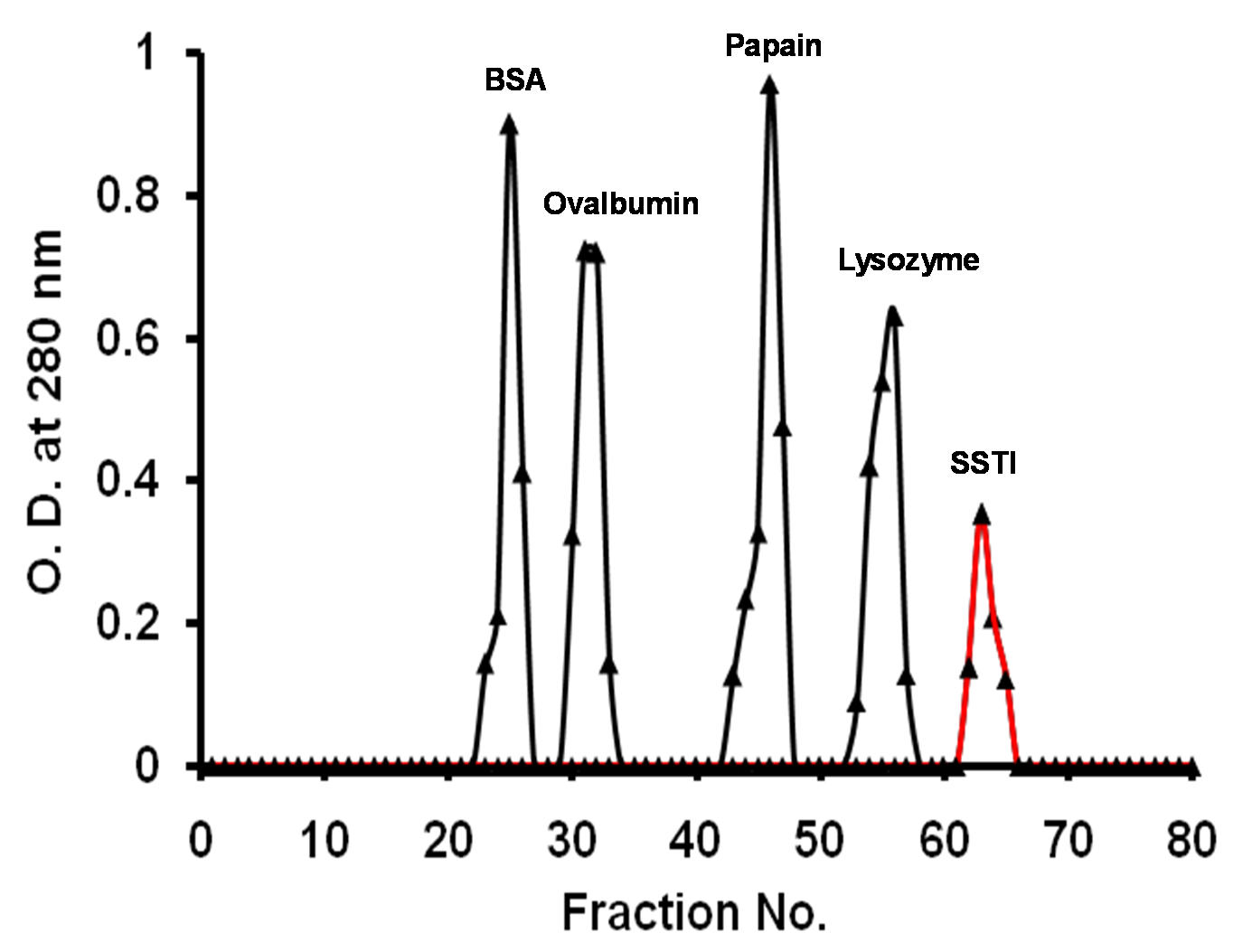
**

**Supplementary Figure S1:** Determine the Molecular mass of SSTI through gel permeation chromatography (Sephadex G-50) using known molecular weight of proteins (Bovine serum albumin, Ovalbumin, Papain, Lyzozyme).

**
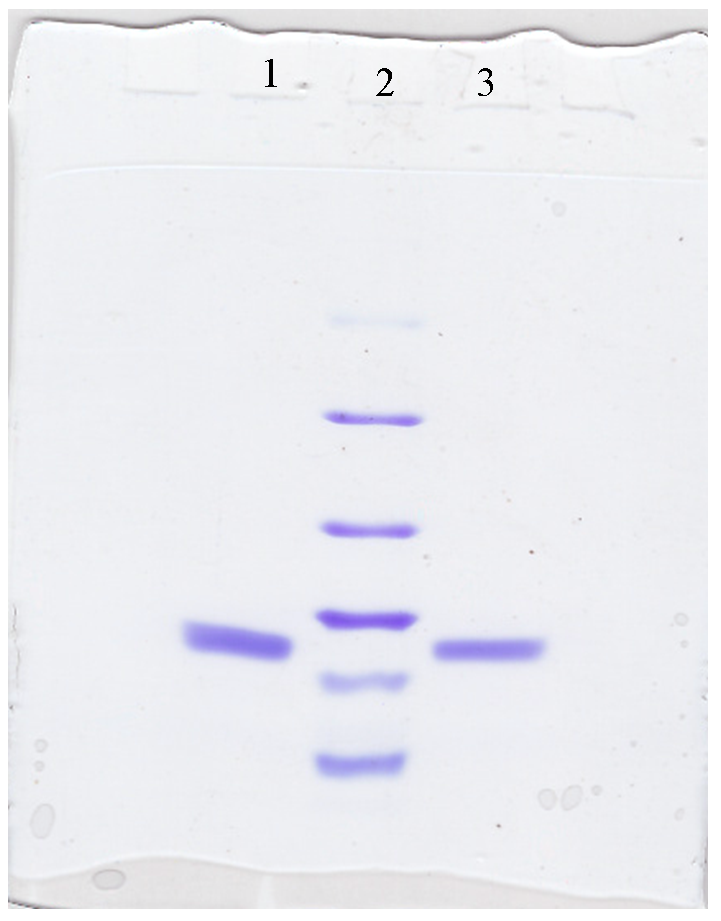
**

**Suplymentry Figure S2A** SDS-PAGE profile (15%), analyses of purified SSTI from, *S. surattense* seeds, stained with Coomassie Brilliant Blue. Lane 1, 3: Purified fraction after cation exchange resin (15 µg and 10 µg respectively) 2: Standard protein markers (10 µg)

**
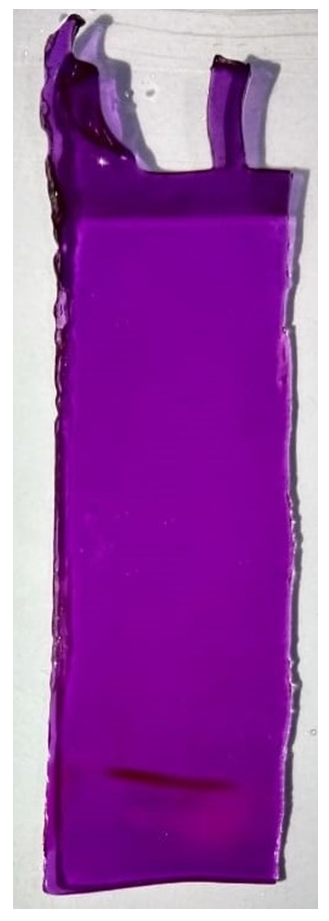
**

**Suplymentry Figure S2B** PAS staining of purified SSTI (10 μg), indicating its glycoprotein nature (12 % SDS-PAGE).
